# Development and comparison of forecast models of hand-foot-mouth disease with meteorological factors

**DOI:** 10.1038/s41598-019-52044-5

**Published:** 2019-10-30

**Authors:** Tao Fu, Ting Chen, Zhen-Bin Dong, Shu-Ying Luo, Ziping Miao, Xiu-Ping Song, Ru-Ting Huang, Ji-Min Sun

**Affiliations:** 1 Key Laboratory of Vaccine, Prevention and Control of Infectious Disease of Zhejiang Province, Zhejiang Provincial Center for Disease Control and Prevention, Hangzhou, China; 2 Yiwu Municipal Center for Disease Control and Prevention, Yiwu, China; 3 West China School of Public Health, Sichuan University, Chengdu, China; 4 Juxian Center for Disease Control and Prevention, Juxian, China; 5 State Key Laboratory of Infectious Disease Prevention and Control, Collaborative Innovation Center for Diagnosis and Treatment of Infectious Diseases, National Institute for Communicable Disease Control and Prevention, Chinese Center for Disease Control and Prevention, Beijing, China; 6 Fengtai Center for Disease Control and Prevention, Beijing, China

**Keywords:** Risk factors, Diseases

## Abstract

Hand-foot-mouth disease (HFMD) is an acute intestinal virus infectious disease which is one of major public health problems in mainland China. Previous studies indicated that HFMD was significantly influenced by climatic factors, but the associated factors were different in different areas and few study on HFMD forecast models was conducted. Here, we analyzed epidemiological characteristics of HFMD in Yiwu City, Zhejiang Province and constructed three forecast models. Overall, a total of 32554 HFMD cases were reported and 12 cases deceased in Yiwu City, Zhejiang Province. The incidence of HFMD peaked every other year and the curve of HFMD incidence had an approximately W-shape. The majority of HFMD cases were children and 95.76% cases aged ≤5 years old from 2008 to 2016. Furthermore, we constructed and compared three forecast models using autoregressive integrated moving average (ARIMA) model, negative binomial regression model (NBM), and quasi-Poisson generalized additive model (GAM). All the three models had high agreements between predicted values and observed values, while GAM fitted best. The exposure-response curve of monthly mean temperature and HFMD was approximately V-shaped. Our study explored epidemiological characteristics of HFMD in Yiwu City and provided accurate methods for early warning which would be great importance for the control and prevention of HFMD.

## Introduction

Hand-foot-mouth disease (HFMD) is an acute intestinal infectious disease which is caused by Enterovirus 71 (EV71), Coxsackie virus A16 (CA16), and other intestinal viruses^1,2^. It is mainly transmitted by direct contact or indirect contact with saliva, nasal mucus or other nasopharyngeal secretions of HFMD patients^3^. The main clinical symptoms include fever, vesicular exanthema on hands, feet, mouths, or buttocks^4^. Most patients recover within 7–10 days without any clinical attention due to its typically mild and self-limiting nature. However, HFMD cases caused by EV71 are more severe and they have severe complications including myocarditis, pulmonary edema, aseptic meningoencephalitis, or even death^5^. Most HFMD patients are infants or children aged ≤5 years old^6^.

HFMD was firstly identified in New Zealand in 1957 and then it was confirmed, especially in Asia-Pacific regions^7–10^. In recent years, HFMD outbreaks were frequently reported in Japan, Malaysia, Singapore, Vietnam and Cambodia^11–16^. In mainland China, about 13.80 million HFMD cases including 130 thousand serious cases and 3300 deaths were reported from 2008 to 2015.

Previous studies reported that HFMD incidence was significantly influences with meteorological factors such as temperature, relative humidity, and so on^6,17–19^.

However, few studies were focus on forecast models of HFMD incidence which is of vital importance for HFMD prevention. Zhejiang is a southeastern coastal province of China and a total of 875945 HFMD cases were identified from 2008 to 2015^20^. Here, we not only analyzed the association between HFMD occurrence and meteorological factors in a city of Zhejiang Province, but also constructed and compared three prediction models for HFMD.

## Materials and Methods

### Data collection

HFMD cases were diagnosed according to “HFMD Control and Prevention Guide” issued by the National health commission of the People’s Republic of China. HFMD cases should be reported to China Information System for Disease Control and Prevention (CISDCP, http://www.cdpc.chinacdc.cn) within 24 h after diagnosis. Information about HFMD cases from 2008 to 2016 in Yiwu City, Zhejiang Province, such as gender, age, occupation, and date of illness onset were obtained from CISDCP. Ethical approval for the study was obtained from the Chinese Center for Disease Control and Prevention Ethics Committee (No. 201214). Informed consent was obtained from all subjects or, if subjects were under 18, from a parent and/or legal guardian. All methods were carried out in accordance with relevant guidelines and regulations and all experimental protocols were approved by Chinese Center for Disease Control and Prevention when HFMD cases were diagnosed in hospitals.

Meteorological data including sunshine duration, monthly precipitation, monthly maximum temperature (T_max_), monthly minimum temperature (T_min_), monthly mean temperature (T_mean_), monthly wind speed (W_speed_), monthly minimum relative humidity (RH_min_), and monthly mean relative humidity (RH_mean_) in Yiwu City were downloaded from the China Meteorological Administration Network (http://data.cma.cn/).

### Statistical analysis

Statistical analysis was performed with the use of R 3.5.0 and Statistical Product and Service Solutions (SPSS 20.0; Chicago, IL). Descriptive statistics were used to analyze the demographic characteristics and seasonal distribution of HFMD in Yiwu City, Zhejiang Province, China. We used Chi square test to compare gender distribution and seasonal distribution of HFMD cases in different years.

The dataset from 2008 to 2015 was used to develop forecast models and the dataset from the dataset of 2016 were used to test the fit of forecast models. Prior to development of forecast models, correlation analysis was conducted to identify collinearity of independent variables.

ARIMA model was constructed as previously described^21^. Briefly, partial autocorrelation function (PACF) and autocorrelation function (ACF) were analyzed to decide the parameters (p, d, q). The optimal model was selected according to Akaike information criterion (AIC).

The number of monthly HFMD cases in the study is over-dispersed (variance/mean = 502.98), so negative binomial model was selected to analyze the relationship of HFMD and meteorological data. Furthermore, quasi-Poisson generalized additive model (GAM) was also selected to develop forecast model as meteorological factors may have a non-linear relationship with HFMD occurrence. These models were also developed and the optimal models were selected as previously described^21^.

In order to compare the agreement between observed data and forecast data of three models, F test was conducted and intraclass correlation coefficient (ICC) was calculated.

## Results

**Figure 1:**
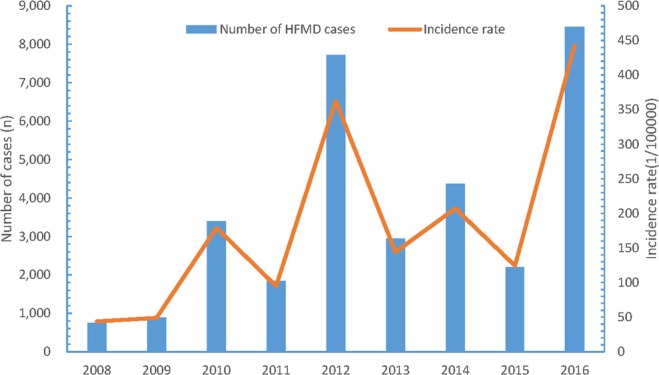
Annual numbers and incidences of HFMD in Yiwu City from 2008 to 2016.

Overall, a total of 32554 HFMD cases were reported and 12 cases deceased in Yiwu City, Zhejiang Province. The number of report HFMD cases from 2008 to 2016 were 747, 886, 3391, 1845, 7724, 2938, 4369, 2196, and 8458, respectively (Fig. 1). Of note, the incidence of HFMD peaked every other year and the curve of HFMD incidence had an approximately W-shape.

About 63.32% HFMD cases were male (20613/32554) and gender distribution of HFMD cases in different years was not similar (χ2 = 41.939, P = 0.000). The numbers of < 1 year old group, 1∼ years old group, 2∼ years old group, 3∼ years old group, 4∼ years old group, 5∼ years old group and > 5 years old group were 4002, 10140, 7668, 5632, 2609, 2170, and 333, respectively. Most HFMD cases were children and 95.76% cases aged ≤5 years old from 2008 to 2016. The majority of HFMD cases were scattered children and kindergarten’s children which accounted for 64.13% and 33.36%, respectively.

**Figure 2:**
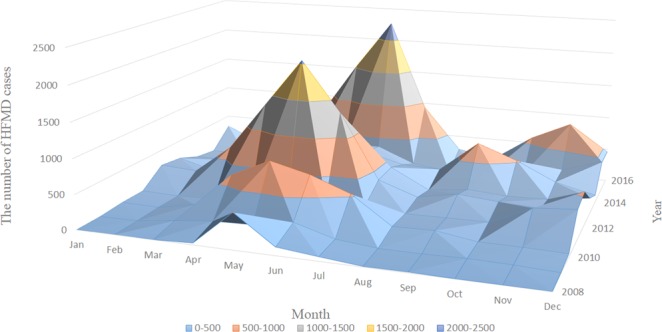
Seasonal distribution of HFMD in Yiwu City from 2008 to 2016.

HFMD cases occurred in every month from January to December, but most cases were reported between April and July (19742/32554) from 2008 to 2016. The month distribution of HFMD cases in different years was not similar (χ2 = 9159.738, P = 0.000). The number of HFMD cases reported in 2008, 2009, 2010, 2012, 2013, 2015 and 2016 peaked in May and June, and that in 2011 peaked in November and December (Fig. 2).

The median of monthly HFMD of 108 months from January 2008 to December 2016 was 181.50 cases and the highest number was 2263 cases. The median values of sunshine duration, Tmin, Tmax, Tmean, monthly precipitation, Wspeed, RHmin, and RHmean were 133.45 h, 15.45 °C, 23.80 °C, 18.80 °C, 122.10 mm, 1.80 m/s, 23.50%, and 69.00%, respectively (Table 1). According to results of correlation analysis, Tmax, Tmin, and Tmean were highest correlated, and RHmin and RHmean were collinearity. So we selected one of Tmax, Tmin, and Tmean, and one of RHmin and RHmean when we constructed forecast models.

**Table 1:** Monthly HFMD occurrence and meteorological factors in Yiwu City from 2008 to 2016.

Variable	P_0_	P_25_	P_50_	P_75_	P_100_
Number of monthly HFMD cases	0	61.50	181.50	402.75	2263.00
Sunshine duration (h)	29.40	103.90	133.45	172.00	311.40
Monthly minimum temperature (°C)	−0.10	7.53	15.45	22.75	27.80
Monthly maximum temperature (°C)	4.90	15.68	23.80	29.65	37.20
Monthly mean temperature (°C)	2.00	11.05	18.80	25.78	32.00
Monthly precipitation (mm)	6.30	64.38	122.10	192.75	504.50
Monthly wind speed (m/s)	1.00	1.70	1.80	1.90	2.30
Monthly minimum relative humidity (%)	12.00	19.00	23.50	31.00	49.00
Monthly mean relative humidity (%)	52.00	64.00	69.00	76.00	91.00

**Figure 3:**
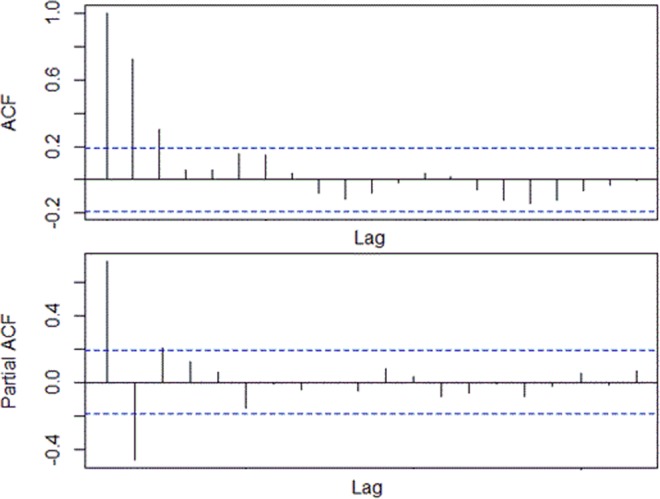
ACF and PACF of ARIMA.

**Figure 4:**
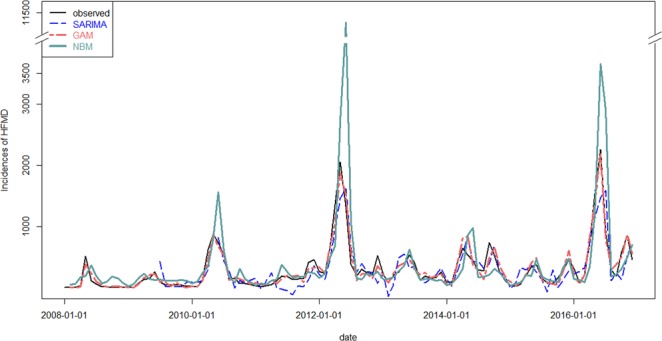
Fit and prediction of three models.

Prior to construction of ARIMA model, results of ADF test indicated time series of monthly HFMD cases was stationary (DF = −3.5224, P = 0.04343) and results of Lung-Box test indicated that the time series was not of random (χ2 = 57.613, P = 0.000). According to the results of ACF, PACF, and AIC, ARIMA (0, 0, 2) × (0, 1, 1)_12_ was selected as the optimal model (Fig. 3). Monthly minimum temperature was significantly associated with the number of monthly HFMD cases. Using the optimal ARIMA model, the predicted numbers of monthly HFMD cases of 2016 in Yiwu City were 229, 265, 325, 729, 1136, 1466, 1593, 109, 245, 130, 511, and 713, respectively. The predicted values were in good agreement with observed data (Fig. 4). ICC of the optimal ARIMA model was 0.878 indicating that this model was excellent (Table 2).

**Table 2:** ICC and F test of the three forecast models.

Model	ICC	95% CI of ICC	F-test value	P-value
NBM	0.834	(0.424, 0.952)	6.030	0.003
ARIMA	0.878	(0.577, 0.965)	8.211	0.001
GAM	0.993	(0.976, 0.998)	147.719	<0.001

Based on the AIC values, the optimal NBM was the following:

$$\begin{array}{rcl}\log ({\mu }_{t}) & = & {\beta }_{0}+as.factor(month)+{\beta }_{1}({\rm{Caset}}1)\\  &  & +\,{\beta }_{2}(Wspeed)+{\beta }_{3}(Wspeedt1)+{\beta }_{4}(Tmean)+{\beta }_{5}(Tmeant1)\end{array}$$

µt, β0, month, Caset1, Wspeed, Wspeedt1, Tmean, Tmeant1 represented the number of predicted HFMD cases during month t, the intercept, the month ordinal, the number of HFMD cases during the previous month, the wind speed, the mean wind speed during the previous month, the mean temperature, and the mean temperature during the previous month, respectively. The values of β2, β3, β4, and β5 were 0.4743, 0.2854, 1.1924, and 3.2772, respectively. In another word, HFMD incidence decreased 52.77% or 71.46% if Wspeed or Wspeedt1 increased a unit, HFMD incidence increased 19.24% or 27.72% if Tmean or Tmeant1 increased a unit.

Using the optimal NBM model, the predicted numbers of monthly HFMD cases of 2016 in Yiwu City were 145, 97, 155, 664, 2429, 4459, 2712, 193, 248, 667, 735, and 341, respectively. The predicted values were in good agreement with observed data (Fig. 4). ICC of the optimal ARIMA model was 0.834 indicating that the model was excellent (Table 2).

Based on the values of deviance explained (%), R square, and GCV principles, the optimal GAM was the following:

$$\begin{array}{c}\log ({\mu }_{t})={f}_{0}+as.factor(month)+ns(Caset1,df=4\,\ast \,9)+ns(Tmean,df=3)\\ \,\,\,\,\,\,\,+\,ns(RHmean,df=4)\end{array}$$

**Figure 5:**
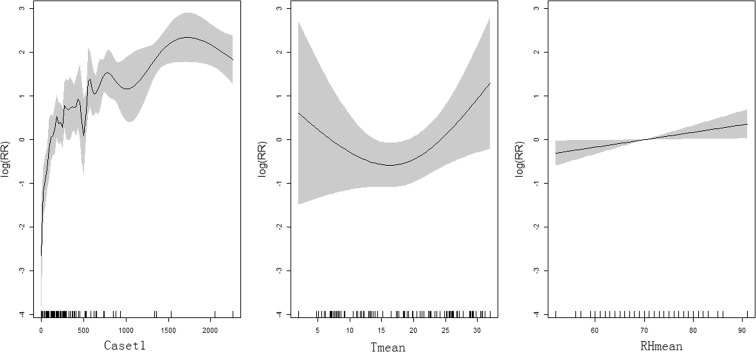
The relationship between HFMD and associated factors.

µt, f0, month, Caset1, and Tmean represented similar variables in NBM. “RHmean” was the monthly mean relative humidity. As shown in Fig. 4, HFMD cases in the previous month and RHmean were positively associated with HFMD incidence. However, the exposure-response curve of monthly mean temperature and HFMD cases occurrence was approximately V-shaped and Tmean showed positive effects on HFMD incidence when it was higher than 17 °C (Fig. 5). Based on the optimal GAM model, the predicted numbers of monthly HFMD cases of 2016 in Yiwu City were 199, 58, 215, 824, 1399, 2180, 821, 289, 387, 499, 865, and 523, respectively. The predicted values were in good agreement with observed data (Fig. 4). ICC of the optimal ARIMA model was 0.993 indicating that the model was perfect (Table 2).

## Discussion

Since HFMD was included in the management of Class C notifiable infectious diseases in mainland China in May 2008, HFMD cases were reported in most provinces of China. A cluster analysis indicated that the incidence rate of HFMD in Zhejiang Province, Hainan Province, Guangxi Province, Shanghai City and Beijing City was higher than that in other provinces^22^. In Zhejiang Province, at least tens of thousands of HFMD cases were reported every year and 212536 HFMD cases were identified in 2014^20^. Yiwu City is located in the center of Zhejiang Province and clusters of HFMD cases have been identified in this city^23^. In this study, we found that the incidence rate of HFMD was upward despite the fluctuations. The results informed that comprehensive measures should be conducted to prevent the increase of HFMD incidence rate.

Similar to other studies, our study indicated that 95.76% HFMD cases aged ≤5 years and most cases were male^24,25^. Decrease immune function, more exposure chance, and genetic susceptibility may contribute this result. Nevertheless, male children aged ≤5 years were the emphasis for control and prevention of HFMD. Most HFMD cases were reported during April and July, but the peak period of different years was slightly different. Notably, the peak period in 2011 was November and December. The reason may be that weather factors of November and December, 2011 were suitable for the growth and transmission of HFMD pathogens. The result suggested that control measures should also be conducted in other months according to the fluctuation of HFMD incidence rate.

Previous studies reported that some meteorological factors were associated with HFMD incidence, but the associated factors were different in different areas. A study in Hefei City, China reported that HFMD occurrence was significantly influenced by extreme precipitation and the effect was the greatest at 6 days lag^26^. Yu G *et al*. reported that high precipitation, extreme temperatures and low-O3 concentration increased HFMD incidence, whereas extremely high wind speed, low PM2.5, low precipitation, and high O3 decreased HFMD incidence in Guilin City, China^27^. Wang P *et al*. found that the year-round temperature and relative humidity, sun duration in winter, and rainfall in summer significantly influenced HFMD incidence^28^. A study in Hong Kong reported that relative humidity, temperature, rainfall, solar radiation and wind speed were associated with HFMD incidence^29^. Moreover, Tian L *et al*. found that mean temperature, relative humidity, wind velocity and sunshine hours were all positively associated with HFMD in Beijing City, but Liu W *et al*. found relative humidity had no relationship with HFMD in Jiangsu Province^24,30^. In our study, NBM and GAM results indicated that monthly mean relative humidity, monthly mean temperature, and wind speed were significantly associated with HFMD. The different risk factors in different areas suggested that different interventions should be conducted in different areas and control measures should be more accurate.

Beside analysis of meteorological factors associated with HFMD incidence, we selected three forecast models using ARIMA, NBM, and GAM to predict HFMD incidence in Yiwu City. During the construction of ARIMA modes, periodic changes, long term trends, and random disturbances were all taken into account. Up to date, ARIMA models have been widely used in infectious diseases prediction including malaria, hemorrhagic fever with renal syndrome (HFRS), and influenza, and so on^31,32^. Our study found that ARIMA was also suitable for the forecast of HFMD incidence.

Due to over dispersion of monthly HFMD cases, NBM was selected to construct prediction model instead of Poisson model in our study. The optimal NBM also had good agreement between prediction values and observed values, but ICC of this model was the least. We found that the prediction was good in months when the number of HFMD cases was small, but the prediction was not good in months when the number of HFMD cases was large. The prediction value was significantly larger than observed value during HFMD peak periods.

GAM can analyze nonnormally distributed data^33^. It can adjust not only nonlinear, nonparametric, and trends, but also confounding effects of seasonality, and weather variables. In our study, we used a quasi-Poisson GAM to analyze relationship between meteorological factors and HFMD and develop HFMD model. ICC of GAM indicated that this model was perfect for prediction of HFMD occurrence. In addition, we found that monthly mean temperature had positive effect on HFMD when it was higher than 17 °C. It suggested that 17 °C of monthly mean temperature could be considered as an alarm value for early warning of HFMD.

In summary, our study not only analyzed epidemiological characteristics of HFMD in Yiwu City but also explored associated meteorological factors and developed three forecast models. All the three models had high agreements between predicted values and observed values, while ICC of GAM was the best. We also identified that temperature, relative humidity, and wind speed were significantly associated with HFMD. The exposure-response curve of monthly mean temperature and HFMD cases occurrence was approximately V-shaped and it showed positive effects on HFMD incidence when it was higher than 17 °C. Our study explored epidemiological characteristics of HFMD in Yiwu City and provided accurate methods for early warning which would be great importance for the control and prevention of HFMD.

## References

[1] Seo D (2018). Estimating the incidence of cases and deaths resulting from hand, foot and mouth disease and its related socioeconomic disease burden in Republic of Korea (2010–2014). Osong Public Health Res Perspect..

[2] Yoshitomi H (2018). Molecular epidemiology of coxsackievirus A6 derived from hand, foot, and mouth disease in Fukuoka between 2013 and 2017. J Med Virol..

[3] Ooi MH (2010). Clinical features, diagnosis, and management of enterovirus 71. Lancet Neurol..

[4] Lai CC (2016). A dynamic model for the outbreaks of hand, foot, and mouth disease in Taiwan. Epidemiol Infect..

[5] Jones E (2018). Outcomes following severe hand foot and mouth disease: a systematic review and meta-analysis. Eur J Paediatr Neurol..

[6] Xing W (2014). Hand, foot, and mouth disease in China, 2008-12: an epidemiological study. Lancet Infect Dis..

[7] Duff MF (1968). Hand-foot-and-mouth syndrome in humans: coxackie A10 infections in New Zealand. Br Med J..

[8] Nguyen NT (2014). Epidemiological and clinical characteristics of children who died from hand, foot and mouth disease in Vietnam, 2011. BMC Infect Dis..

[9] Sasidharan CK (2005). Hand-foot-and-mouth disease in Calicut. Indian J Pediatr..

[10] Zhang Y (2010). An emerging recombinant human enterovirus 71 responsible for the 2008 outbreak of hand foot and mouth disease in Fuyang city of China. Virol J..

[11] Ang LW (2009). Epidemiology and control of hand, foot and mouth disease in Singapore, 2001–2007. Ann Acad Med Singapore..

[12] Biswas T (2012). Enterovirus 71 causes hand, foot and mouth disease outbreak in Cambodia. Natl Med J India..

[13] Chua KB (2011). Hand foot and mouth disease due to enterovirus 71 in Malaysia. Virol Sin..

[14] Fujimoto T (2002). Outbreak of central nervous system disease associated with hand, foot, and mouth disease in Japan during the summer of 2000: detection and molecular epidemiology of enterovirus 71. Microbiol Immunol..

[15] Ho M (1999). An epidemic of enterovirus 71 infection in Taiwan. Taiwan Enterovirus Epidemic Working Group. N Engl J Med..

[16] Khanh TH (2012). Enterovirus 71-associated hand, foot, and mouth disease, Southern Vietnam, 2011. Emerg Infect Dis..

[17] Huang Y (2013). Effect of meteorological variables on the incidence of hand, foot, and mouth disease in children: a time-series analysis in Guangzhou, China. BMC Infect Dis..

[18] Lin H (2013). Short-term effect of El Nino-Southern Oscillation on pediatric hand, foot and mouth disease in Shenzhen, China. PLoS One..

[19] Onozuka D (2011). The influence of temperature and humidity on the incidence of hand, foot, and mouth disease in Japan. Sci Total Environ..

[20] Wu C (2016). Epidemiology of hand foot and mouth disease in Zhejiang, 2008–2015. Dis Surveillance..

[21] Sun JM (2018). Forecast of severe fever with thrombocytopenia syndrome incidence with meteorological factors. Sci Total Environ..

[22] Zhang YJ (2015). Epidemic characteristics of hand, foot and mouth disease in mainland China, 2008-2010: a cluster analysis. Chin. J Public Health..

[23] Cai J (2014). Temporal-spatial scan clustering analysis on hand-foot-mouth disease in Zhejiang Province, 2008-2013. Chin J Prevent Med..

[24] Liu WD (2015). Spatiotemporal dynamics of hand-foot-mouth disease and its relationship with meteorological factors in Jiangsu Province, China. PLoS One..

[25] Jin Y (2012). Epidemiology of hand, foot and mouth disease in mainland of China, 2011. Dis Surveillance..

[26] Cheng J (2014). Associations between extreme precipitation and childhood hand, foot and mouth disease in urban and rural areas in Hefei, China. Sci Total Environ..

[27] Yu GQ (2019). Short-term effects of meteorological factors and air pollution on childhood hand-foot-mouth disease in Guilin, China. Sci Total Environ..

[28] Wang P (2017). Seasonal modeling of hand, foot, and mouth disease as a function of meteorological variations in Chongqing, China. Int J Biometeorol.

[29] Wang P (2016). Hand, foot and mouth disease in Hong Kong: a time-series analysis on its relationship with weather. PLoS One..

[30] Tian L (2018). Spatio-temporal analysis of the relationship between meteorological factors and hand foot-mouth disease in Beijing, China. BMC Infect Dis..

[31] Loha E (2010). Model variations in predicting incidence of Plasmodium falciparum malaria using 1998–2007 morbidity and meteorological data from south Ethiopia. Malar J..

[32] Liu Q (2011). Forecasting incidence of hemorrhagic fever with renal syndrome in China using ARIMA model. BMC Infect Dis..

[33] Hastie, T. J. *et al*. Generalized additive models. *Chapman & Hall/CRC* (1990).

